# Efficacy and safety of acupuncture in postoperative ileus after gynecological surgery: A protocol for system review and meta-analysis of randomized controlled trials

**DOI:** 10.1097/MD.0000000000024342

**Published:** 2021-02-05

**Authors:** Yi Guo, Xianglu Kong, Qiuyu Cao, Yin Li, Yuzhuo Zhang, Jieming Huang, Kunyin Li, Yongge Guan

**Affiliations:** a Guangzhou University of Chinese Medicine, Guangzhou; b Jiande hospital of integrated traditional Chinese and Western Medicine, Hangzhou; c The first affiliated hospital of Guangzhou University of Chinese Medicine, Guangzhou; d The third affiliated hospital of Guangzhou University of Chinese Medicine, Guangzhou, China.

**Keywords:** acupuncture, gynecological surgery, postoperative ileus, protocol, systematic review

## Abstract

**Background:**

Acupuncture is widely used in treatment of postoperative ileus (POI), but the safety and efficacy of acupuncture in POI after gynecological surgery still lack of evidence-based basis.

**Methods:**

PubMed, CINAHL, EMBASE, Web of science, Google Scholar, Wangfang database, Chinese Biomedical Literature Database (SinoMed), Chinese Science and Technology Periodical Database, and China National Knowledge Infrastructure database will be searched until December 31, 2020. Two independent investigators will screen the relevant randomized controlled trials from Data one by one by using prespecified criteria. The relevant data from included studies will be extracted and analyzed by using RevMan V.5.3 software. Quality of the included studies will be estimated by using the Cochrane Collaboration risk of bias tool, and publication bias will be assessed by using Egger test and Begg test. In addition, quality of evidence will be evaluated by using Grading of Recommendations Assessment, Development, and Evaluation.

**Results:**

We will analyze the effect of acupuncture on time to first flatus and time to bowel sound recovery as the primary outcomes of this review. Meanwhile, frequency of bowel sounds, time to defecation, time of hospital stay, biochemical indicators related to gastrointestinal motility, inflammation factors, responder rate, and adverse events for patients receiving gynecological surgery.

**Conclusion:**

Our findings will benefit researchers and provide reference for the treatment and prevention of POI for the patients undergoing gynecological surgery.

## 1 Introduction

Postoperative ileus (POI) is the condition where the motor activity of the bowel is impaired,^[1]^ which serves as a frequent complication occurring immediately after all kinds of surgeries, such as colon and rectal surgery,^[2]^ radical gastrectomy for gastric cancer,^[3]^ spinal operation,^[3]^ and gynecological surgery.^[4]^ It always manifests as abdominal discomfort, pain, nausea, vomiting, and distension,^[5]^ which will delay the rehabilitation after surgery, prolong hospital stay, increasing cost, and even associated the increases in both morbidity and mortality.^[6]^ There are various risk factors believed to be associated with the development of POI, including operative time and difficulty, gastrointestinal inflammatory response, anesthesia, and use of opioid analgesics.^[7]^ As is reported, the incidence of POI following gynecological surgery ranges from 5% to 25%, especially after major surgeries.^[8]^ Furthermore, it is suggested that incidence of POI shows no significantly difference among 3 different gynecological procedures through abdominal, laparoscopic, and vaginal approaches.^[9]^ Due to the relatively high prevalence and unclear mechanism, POI has been a major clinical problem and a challenge for the treatment worldwide,^[10]^ which not only strongly affects the postoperative recovery, but also brings a heavy burden on family and society. Apart from improving surgical programs and skills, it is also crucial and urgent to develop a safe and effective approach for the prevention and treatment of POI.

Acupuncture, as an integral part of traditional Chinese medicine with thousands of years history, has dual regulatory effects on gastric motility, comprising of promoting gastrointestinal peristalsis in subjects with low initial gastric motility, and inhibiting peristalsis in subjects with active initial motility.^[11]^ Moreover, it is proved that acupuncture is a potential strategy to treat gastrointestinal dysfunction diseases.^[12]^ Considering the few side effects, it is widely used to decrease incidence of POI and improve its symptoms. In recent years, there are growing number of studies conducted to explore the benefits of acupuncture for POI. In the previous meta-analysis, it was suggested that acupuncture ameliorates POI with significant heterogeneity,^[13]^ indefinite conclusion,^[14]^ or restriction on sort of disease (colorectal cancer).^[15]^ Gynecological surgery has its own characteristics, including surgery approaches and gynecological disease itself, which might result a different conclusion. However, until now, the safety and efficacy of acupuncture in POI after gynecological surgery are still lack of evidence-based basis. Consequently, this systematic review and meta-analysis is designed to examine the safety and efficacy of acupuncture in the POI for the patients undergoing gynecological surgery.

## 2 Methods

The present meta-analysis will be carried out in accordance with the Preferred Reporting Items for Systematic Review and Meta-Analyses .^[16]^ The protocol of this review has been registered at the Open Science Framework (Available at: https://osf.io/ym8s9), and the registration DOI of this study is 10.17605/OSF.IO/J3AH9. Any amendments to this registered protocol will be submitted to the database along with the explanations for every changes.

### 2.1 Selection criteria

The detailed eligibility criteria are established based on the PICOS approach (patients, intervention, comparisons, outcomes, and study design type).

#### 2.1.1 Patients

We will include the studies conducted in adult patients of either sex who receiving gynecological surgery in the present review and meta-analysis. The gynecological procedures, whether abdominal, laparoscopic, and vaginal approaches, should be considered.

#### 2.1.2 Intervention

We define acupuncture including traditional manual acupuncture and electroacupuncture as the experimental intervention, whereas studies involving scalp acupuncture, wrist-ankle acupuncture, transcutaneous electroacupuncture, or ear acupuncture will be excluded. We will include the studies in which acupoints are only stimulated by traditional acupuncture needling, hence the studies regarding acupressure, laser acupuncture, or dry needling will be excluded.

#### 2.1.3 Comparators

Studies in which experimental group receiving acupuncture was compared to control group receiving other therapy or without intervention will be considered. In order to understand the effect of acupuncture, the following 4 comparisons will be included in this study: acupuncture versus basic or conventional treatment; acupuncture combined with basic or conventional treatment versus basic or conventional treatment used alone; acupuncture versus other treatment; acupuncture versus placebo or sham acupuncture.

#### 2.1.4 Outcome measures

We will consider time to first flatus and time to bowel sound recovery as the primary outcomes of this review. Meanwhile, frequency of bowel sounds, time to defecation, time of hospital stay, biochemical indicators related to gastrointestinal motility including motilin, gastrin, and vasoactive intestinal peptide, inflammation factors including interleukin 1β and white blood cell, responder rate, and adverse events will be reported as secondary outcomes.

#### 2.1.5 Study design

We will collect all the relevant published clinical randomized controlled trials (RCTs) investigating the efficacy of acupuncture on POI for the patients receiving gynecological surgery. The clinical RCTs published in a peer-reviewed journal will be included, and no restriction on language will be imposed. However, the articles including studies without full-text, duplicate articles, unpublished literatures, no sufficient outcomes related to POI, observational studies, case series, animal studies, conferences abstract, letters, comments, and reviews will be excluded.

### 2.2 Search strategy

Paired 2 investigators (Yi Guo and Xianglu Kong) will independently search the PubMed, CINAHL, EMBASE, Web of science, Google Scholar, Wangfang database, Chinese Biomedical Literature Database (SinoMed), Chinese Science and Technology Periodical Database, and China National Knowledge Infrastructure database to identify the potentially relevant articles from inception to December 31, 2020. A keyword such as “acupuncture”, “postoperative ileus”, “randomized”, “randomized controlled trial”, etc will be used to search without restrictions on language or sort of disease. A detailed search strategy for CINAHL is listed in Table 1.

**Table 1:** Search strategies for CINAHL database.

No.	Query
#1	MeSH descriptor: [postoperative ileus] explode all trees
#2	((((((Postoperative gastrointestinal motility disorder) or (postoperative gastrointestinal motility disorder))) or (postoperative gastrointestinal function recovery)) or (postoperative gastrointestinal dysfunction)) or (postoperative ileus)) or (postoperative gastrointestinal function):ti, ab, kw (word variations have been searched)
#3	MeSH descriptor: [electroacupuncture] explode all trees
#4	(Electroacupuncture):ti, ab, kw (word variations have been searched)
#5	MeSH descriptor: [acupuncture] explode all trees
#6	(Acupuncture):ti, ab, kw (word variations have been searched)
#7	#1 or #2
#8	#3 or #4 or #5 or #6
#9	#7 and #8

### 2.3 Study selection and data extraction

**Figure 1:**
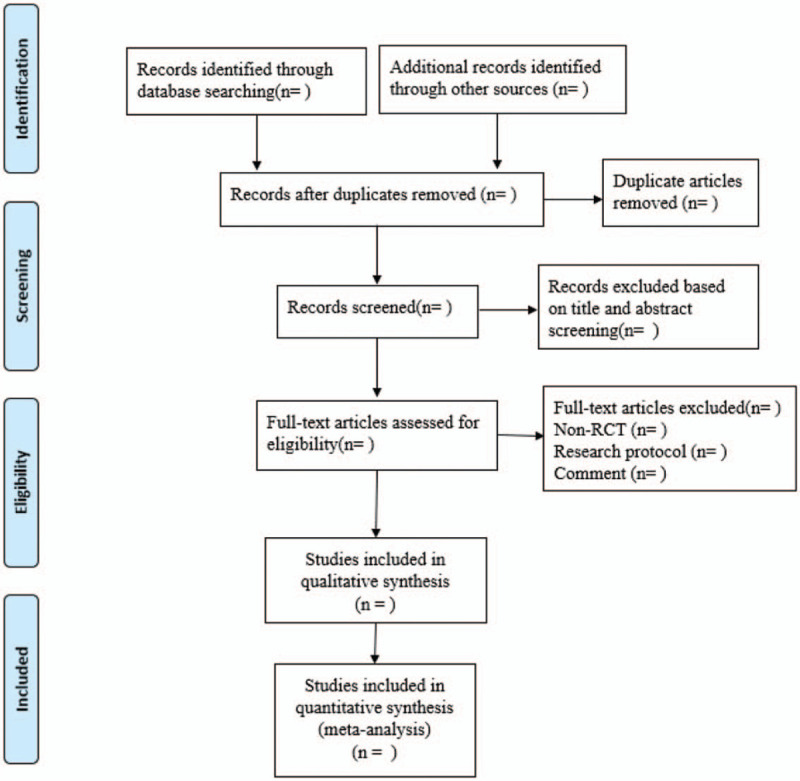
Flow chart of the search process.

In this study, 2 investigators will screen all the literatures separately (Yi Guo and Xianglu Kong). Literatures will be preliminarily examined by screening the title and abstract one by one. Then, full-text articles will be downloaded and checked again detailedly, and included studies will be obtained by using prespecified criteria. The relevant information will be extracted from the included articles, including countries, publication year, age and gender of patients, disease, type of surgery, study design, blind method, intervention type, acupoints, duration of intervention, sample size, outcomes, safety, drop out, and advent events. Any disagreements for literature screening and data extraction will be resolved by discussion, or consultation with a third investigator (Kunyin Li) until final consensus is reached. A Preferred Reporting Items for Systematic Review and Meta-Analyses flow chart of the selection procedure is illustrated in Figure 1.

### 2.4 Quality assessment

The quality of the included RCTs will be independently assessed by 2 reviewers (Yi Guo and Yuzhuo Zhang) according to the Cochrane Collaboration recommendations.^[17]^ The literature will be estimated from 7 aspects: sequence generation, allocation concealment, blind of participants and personnel, blind of outcome, incomplete outcome data, selective reporting, and other biases. The risk of bias will be judged on 3 levels: high, unclear, and low. A primary reviewer will be consulted to resolve any discrepancies.

### 2.5 Data synthesis and analysis

The meta-analysis of the included studies will be done by using the review manger 5.3 software, and illustrated by the forest map intuitively. Uncertainty will be expressed with 95% confidence intervals. Standard mean differences or mean differences will be used to analyze continuous outcomes. Dichotomous data will be described as odds ratios associated with 95% CI. Heterogeneity will be assessed by using Cochran Q-test and I^2^ index.^[18]^*P*<.10 (Cochran Q-test) and I^2^ statistic less than 50% indicate an acceptable heterogeneous so that a random effects models will be employed; otherwise, a fixed effects models will be selected for the meta-analysis, and we will explore the reasons for substantially heterogeneous including conducting sensitivity analysis and subgroup analysis. Subgroup analysis will be performed according to acupuncture and surgery type. *P*-values<.05 is considered to be a statistically significant.

### 2.6 Assessment of reporting biases

Begg and Egger tests will be chosen to evaluate publication bias.^[19]^ When a *P*-value<.05 in Egger test or Begg test is believed to indicate a statistically significant publication bias.

### 2.7 Confidence in cumulative evidence

The Grading Of Recommendations, Assessment, Development, and Evaluation; version: 3.6) approach is widely used to estimate the quality of evidence.^[20]^ In this meta-analysis, the evidence will be graded as 4 levels: high, moderate, low, or very low. Disagreements will be addressed by discussion or consultation for a third reviewer until a consensus achieved.

## 3 Discussion

Gynecological surgery is one of the most common surgery, and often used to treat gynecological diseases, while POI after gynecological surgery cannot be neglected because of its many bad effects. However, despite its common occurrence, POI is still viewed as an ongoing conundrum. With the continuous exploration for prevention and treatment of POI, a variety of strategies was developed to reduce POI, including chewing gum, alvimopan, avoidance of opioid, and minimally invasive surgery, but none of these methods could be completely successful in shortening the duration of POI.^[21]^ Although acupuncture was reported as a potential method to improve POI after gynecological surgery in previous studies,^[22,23]^ its effect on the improvement of pain and function, evidence-based basis remains unknown.

This study will be the first systematically review and meta-analysis to examine the efficacy and safety of acupuncture on POI for the patients receiving gynecological surgery. The results will demonstrate whether acupuncture should be recommended explicitly for the treatment of POI after gynecological surgery. The meta-analysis will benefit researchers and provide reference for the treatment and prevention of POI for the patients undergoing gynecological surgery.

## Author contributions

**Conceptualization:** Yi Guo, Xianglu Kong, Yongge Guan.

**Data curation:** Yi Guo, Xianglu Kong.

**Formal analysis:** Qiuyu Cao and Jieming Huang.

**Investigation:** Yin Li, Yuzhuo Zhang, and Jieming Huang.

**Methodology:** Yi Guo and Xianglu Kong.

**Review sponsor:** Yongge Guan and Kunyin Li.

**Software:** Yin Li, Qiuyu Cao.

**Writing – original draft:** Yi Guo.

**Writing – review & editing:** Yuzhuo Zhang.
